# Dietary Assessment on a Mobile Phone Using Image Processing and Pattern Recognition Techniques: Algorithm Design and System Prototyping

**DOI:** 10.3390/nu7085274

**Published:** 2015-07-27

**Authors:** Yasmine Probst, Duc Thanh Nguyen, Minh Khoi Tran, Wanqing Li

**Affiliations:** 1 School of Medicine, University of Wollongong, Wollongong, NSW 2522, Australia; 2 School of Computing and Information Technology, University of Wollongong, Wollongong, NSW 2522, Australia; E-Mail: dtn156@uowmail.edu.au; 3 Faculty of Information Technology, University of Science Ho Chi Minh City 70000, Vietnam; E-Mail: minhkhoitran2@gmail.com; 4 School of Computing and Information Technology, University of Wollongong, Wollongong, NSW 2522, Australia; E-Mail: wanqing@uow.edu.au

**Keywords:** mHealth, food record, image processing, pattern recognition, food image

## Abstract

Dietary assessment, while traditionally based on pen-and-paper, is rapidly moving towards automatic approaches. This study describes an Australian automatic food record method and its prototype for dietary assessment via the use of a mobile phone and techniques of image processing and pattern recognition. Common visual features including scale invariant feature transformation (SIFT), local binary patterns (LBP), and colour are used for describing food images. The popular bag-of-words (BoW) model is employed for recognizing the images taken by a mobile phone for dietary assessment. Technical details are provided together with discussions on the issues and future work.

## 1. Introduction

Assessment of dietary intake is a process vital to dietetic care across different disciplines and specialties of practice. As a fundamental skill taught during early dietetic training, the manual process of conducting a dietary assessment with a participant is inherently flawed due to various forms of bias depending on the method of assessment being applied [1] in addition to the format of the assessment. Traditionally, assessments were completed using a paper-and-pen format to record *usual* intake through diet history interview, repeated 24-h recall and food frequency questionnaire and are often impacted by memory and cognition of the person recalling their intake. In addition, the time burden of the process and the literacy of the target group for which dietary intake data is required are also factors that affect the data being recorded. This is particularly evident in assessing the intakes of children where the age of the child plays an important role on the method employed. Whether the recall is obtained from the child who consumed the food or whether the recall is provided by the child’s carer or parents, may have impact on the accuracy of what has been consumed [2]. With the combination of these challenges and technological advances, many dietary assessment methods have been partially or fully automated in an attempt to reduce bias, ease the burden for the participants and also streamline the steps applied within each method [1].

Automation of dietary assessment appears to have begun during the 1960s with early movements towards computerized processing of intake data [3]. Along with the move to the computerized analysis of the nutrients, there has been an expansion to the automation of the intake assessment process itself. This initially began with the use of software on a standalone desktop computer and later advanced to interactive processes through the Internet [1]. Today, web-based dietary recalls are not uncommon in large cohort studies due to their efficiency and ability to streamline processes within one study time point [3]. The automated version of the 24-hour recall for example minimizes the need for an interviewer by employing the use of on-screen avatars [4] and probing questions to guide the participants through the recall. This change has also resulted in reduced resource requirements overall allowing it to be implemented with a very large number of participants.

Contrary to this, the most common method of dietary assessment used within the randomized controlled trials remains as that of the food record or food diary [5], a record of *actual* food intake. This method places increased burden on the person completing the assessment and may involve a process of estimating or measuring the food portion size after recording the name of the food item that has been consumed. The method is retrospective in nature and due to the participant burden, modifications made to the intake during the recording period often occur with a longer recording periods resulting in greater bias or underestimation of intake [6]. Automation of the food record method aims to address this issue. More recently, the food record method has shifted to portable devices such as Tablets and Smartphones [1]. This shift should not simply be thought of as the creation of data collection forms on a phone but rather be considered as essential changes to the method within which data collection occurs. During the past decade research in this area has been expanding rapidly. Although there are many applications (apps) available for download to a Smartphone, the credibility of these apps remains questionable. Key groups in the United States have developed credible, evidence-based applications used within the research setting [1]. The Technology Assisted Dietary Assessment (TADA) project, for example, has employed a process of image segmentation to accurately detect food items, with an initial prototype trialed on iPhones/iPods. This group reported challenges of detecting colour and texture [7] and employed the use of a fiducial marker to assist with the accuracy of the detection process. Illumination and the angle of the photos being taken have been further noted as concerns [8]. The Bag-of-Words model, used as the basis for the prototype described in this paper, was also used by the TADA team. Also using image recognition, the Food Intake Visual and voice Recognizer (FIVR) project similarly used a fiducial marker to assist with the image recognition. Contrasting to the TADA project, which initially used text to assist with image tagging, FIVR used voice for this process, and in addition, it relied on the user’s descriptions (text or voice) to identify the food in the photograph [9]. While the above tools aim to automate the assessment, notable progress has also been made with the Remote Food Photography Method (RFPM), which uses a semi-automated food photograph classification approach. The RFPM is focused on portion size of the food items and uses bilateral filtering to reduce the noise in the images taken [10]. User training is required in order to ensure that photographs are captured correctly [11].

This paper describes the development of a baseline prototype of an automated food record by using image processing and pattern recognition algorithms. To the knowledge of the authors, the automated food record described in this paper is the first Australian developed prototype of this nature. The described prototype supports automation of the food recording and recognition. The prototype facilitates an extension to determine the portion size of the food and finally all data can be easily matched to food composition databases. Determination of food portion is crucial for nutrient calculations and automation of this process can reduce the need for manual calculations from the recognized foods. The focus of this paper is to provide a comprehensive overview of a baseline prototype with the function of image recognition and addresses the challenges that are potentially faced when moving an algorithm from a laboratory-based test environment [12] to a user-based application.

The baseline prototype also adopts the Bag-of-Words model for recognition due to computational efficiency and promising results obtained recently [11,16]. Many challenging issues mentioned above are dealt by the careful selection of features. However, the work differs from the previous studies [11,16] in the following dimensions. Firstly, the system does not require any fiducial markers or addition user annotation such as text or voice. Secondly, the system aims to recognize multiple foods in a single image, *i.e*., more than one food type can be present in an input image. Our system is thus more realistic in practice. Thirdly, we are also simultaneously deploying the food image recognition on mobile phones. This enables us to investigate the practical factors of the proposed dietary assessment prototype including the discriminative power of each feature type, the combination of features to particular food categories and the computational speed of various interest point detectors.

## 2. Experimental Section: Image Classification Using Bag-of-Words (BoW) Model

The BoW model was originally devised for text classification [13]. In the model, a text document is encoded by a histogram representing the frequency of the occurrence of codewords. The codewords are predefined in a discrete vocabulary referred to as a codebook. The codeword histograms obtained from training samples (small collections of text documents) are used to train a discriminative classifier, e.g., Support Vector Machine (SVM) [14]. The trained classifier is then used to classify test documents (larger collections of text).

To automate the food record, the BoW model was applied for classification of food images captured using a person’s mobile phone. In this task, the features of an image are used as codewords. The images employed in this study, are the photographs of foods recorded as part of the food record method. Rather than recording the text-based name of the food risking poor quality detail about the food due to long food selection lists, incorrect spelling or typographical errors, a photograph can provide additional details about the foods. Using photographs also provides the potential to minimize the burden on the person completing the dietary assessment. The food image classification method in this study was implemented in C++ programming language. The training phase and evaluation of the food image recognition were conducted on a desktop computer using Microsoft Windows 7.0. The prototype was deployed on a Smartphone using the Android mobile operating system.

Image classification using the BoW model requires feature selection, codebook creation, discriminative training. In the following sections, each of these components is described in further detail.

### 2.1. Feature Selection

For the purpose of image recognition, the features are used to describe the visual properties of the foods in the photographs. In this study, we investigated three common types of features including Scale Invariant Feature Transformation (SIFT) [15], Local Binary Pattern (LBP) [16], and Colour [17]. *SIFT* [15] is used to describe the local *shape* of visual objects. It is constructed by a histogram of the orientations of edges in the food photographs. *LBP* [16] is used to capture the *texture* information of the foods. The LBP is known for its simplicity in implementation, low computational complexity and robustness under varying illumination conditions. *Colour* [17] plays an important role in food image classification, e.g., the red colour of a tomato is useful to distinguish it from an apple sharing a similar shape. In our experiments, we quantise each colour channel (Red, Green, and Blue) of an image pixel into four bins (intervals). The colour features of an image then can be constructed as the histogram of the colour of all pixels in that image.

### 2.2. Codebook Creation

Suppose that there are N food categories $C_{1},C_{2},\ldots ,C_{N}$ (e.g., carrots, muscle meat, *etc.*) and A is the set of training images. The SIFT interest point detector of Lowe [15] was invoked to detect interest points on the training images of A. SIFT [14] and LBP [15] features were then extracted at interest points. Let $v_{p}$ be the D-dimensional features extracted at interest points p. These features were then clustered into K groups using a K-means algorithm. Idendennn clustering, the dissimilarity (based on distance) between two features was computed using a metric, e.g., $\chi^{2}$ distance (as in our implementation). Equation (1) outlines the dissimilarity between features as follows,

(1) $$\left(v_{p},v_{q}\right)=\frac{1}{2}\overset{D}{\underset{i=1}{\sum }}\frac{{\left[v_{p}\left(i\right)-v_{q}\left(i\right)\right]}^{2}}{v_{p}\left(i\right)+v_{q}\left(i\right)}$$

where $v_{p}\left(i\right)$ is the i-th element of $v_{p}$.

Completion of this step resulted in a codebook $G=\left\{w_{1},\;w_{2},\;\ldots ,w_{K}\right\}$ in which codewords $w_{i}$ were considered to be the centres of the *i*’th clusters.

### 2.3. Discriminative Training

To classify N food categories, N binary classifiers $f_{1},f_{2},\ldots ,f_{N}$ were used. Each classifier $f_{i}$ classified a given test sample (food image in this context) into two classes: $C_{i}$ or non-$C_{i}$. Given a food image and codebook G, the SIFT interest point detector was used to detect a set of interest points from the food image. Let p and $v_{p}$ be an interest point and the feature extracted at p. The best matching codeword w(p)∈G was determined as outlined in Equation (2),

(2) $$w\left(p\right)=\underset{w_{i}\in G}{\text{argmin}}\;d\left(v_{p},\;w_{i}\right)$$

where $d\left(v_{p},\;w_{i}\right)$ is defined in Equation (1).

**Figure 1:**
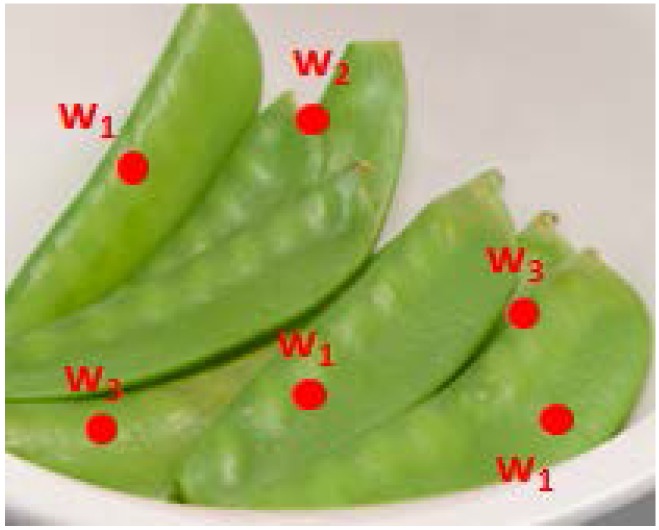
An image of snowpeas showing interest points shown as red points and the corresponding best matching codewords labelled w_1_, w_2_, w_3_.

Figure 1 provides an example of describing a food image by codewords. In this figure, the red points are SIFT interest points, where $w_{1},w_{2},w_{3}$ are the best matching codewords of the features extracted at each of these interest points. The food image was then encoded by a histogram of occurrence of the codewords. Such histograms were collected from all training images of the food category *i*’th and from the training images of other categories to train the classifier $f_{i}$. In our implementation, SVMs were used as classifiers for $f_{i}$.

### 2.4. Testing

Given a test image, I, similar to the training phase, the histogram of the occurrence of the codewords obtained on I was computed and denoted as h(I). Let $f_{i}\left(h\left(I\right)\right)$ be the classification score of the trained classifier $f_{i}$ applied on h(I). If the image I contains only one food category, this food category was determined as shown in Equation (3)

(3) $$C_{i}\;\text{if }f_{i}=\;\underset{j\in \left\{1,\ldots ,N\right\}}{\max}f_{j}\left(h\left(I\right)\right)$$

If I contains more than one food category (such as for a meal on a plate), the food category $C_{i}$ was considered to be present in I if $f_{i}\left(h\left(I\right)\right)>\epsilon$, where ϵ is a user-defined threshold, referred to as recognition sensitivity.

## 3. Results

The SIFT interest point detector took on average approximately 74 seconds for an image captured by a smartphone’s camera. To speed-up the interest point detector, input images were resized by a factor of two if either the width or height of the input image was over 2000 pixels. Through the experiments it was found that by reducing the image’s size, the speed of the interest point detector could be improved and also the number of interest points overall could be reduced. In the experiments, the detection of interest points on the resized images took approximately 20 s. Note that the time also depends on the computing resource available in the Smartphone. Different implementations of the SIFT interest point detector outlined by Lowe [15] were also trialed. Table 1 shows the times taken by those interest point detectors.

**Table 1:** Processing time of interest point detectors.

Interest Point Detector	Processing Time (s/image) ^†^
Original interest point detector [9]	74
ezSift	14
openCV	60–65
zerofog	15–20

**^†^** Obtained without resizing the input image.

The ezSift interest point detector was found to be the fastest detector with less than 15 s. The number of interest points generated by this detector was less than that generated by other detectors, likely due to a lower number of scales used in the ezSift detector. However, the number of detected interest points was usually sufficient for recognition as this has been found empirically. The study also found that the zerofog had been optimized using openmp to run on parallel processors.

To describe the appearance of food images, three codebooks corresponding to three different feature types were created for each food category. The codebook size (*i.e*., the number of codewords) for the SIFT, LBP, and colour feature was 100, 40, and 50, respectively. To combine the three features, for each food image, three histograms of codewords corresponding to the three codebooks were concatenated to form a longer histogram. Linear SVMs [18] were employed as classifiers.

**Figure 2:**
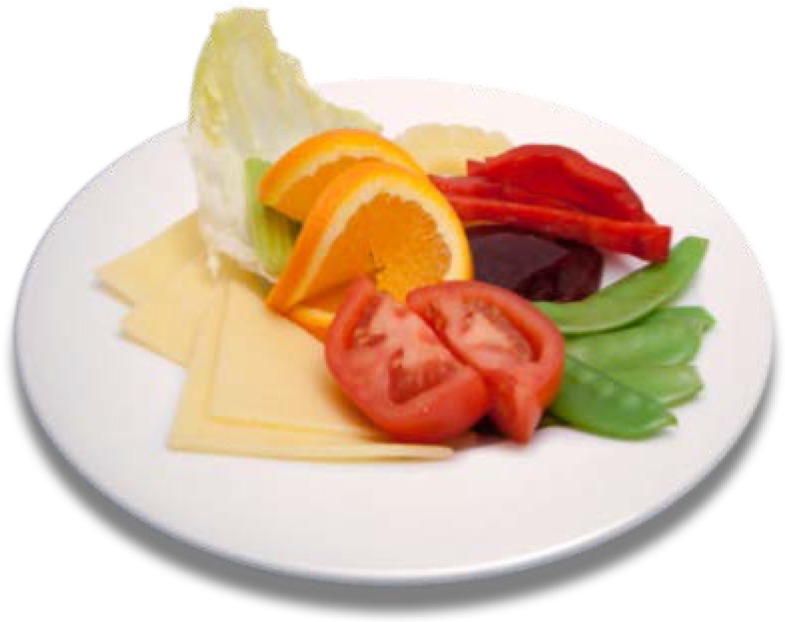
An example of a food image containing five food categories: cheese, tomato, oranges, beans, and carrots.

The food image classification method was evaluated on a newly created dataset [19,20,21]. Table 2 summarises the dataset used in the evaluation. Note that in this dataset, one food image may contain more than one food category (see Figure 2). The dataset was organised so that the training sets (used during codebook creation) and test sets were separated.

**Table 2:** Summary of the dataset used for training and testing of the food image recognition method. Positive images of a food category indicate the number of images contained in each food category.

Food Category	Positive Training Images, *n*	Test Images, *n*
Beans	4	18
Carrots	7	47
Cheese	4	53
Custards	5	64
Milk	4	44
Muscle meat	7	34
Oranges	8	61
Peas	5	13
Tomato	6	24
Yoghurt	6	52

Since more than one food category could be contained in a food image, as would be consumed in real life, a food category $C_{i}$ was considered to be contained in the food image I if $f_{i}\left(h\left(I\right)\right)>\epsilon$. Thus, the classification (recognition) performance was investigated by varying the threshold ϵ. Let M be a set of test images, $M_{i}$ be a subset of M and contain the food category $C_{i}$. For a given ϵ, let $M_{i}^{R}\left(\epsilon \right)\;M$ be the set of images whose classification score is greater than ϵ, *i.e*., $M_{i}^{R}\left(\epsilon \right)$ is the set of images recognised as containing instances of $C_{i}$. The recognition performance associated with ϵ of the food category $C_{i}$ was represented by $r_{i}\left(\epsilon \right)$ and defined in Equation (4) as follows,

(4) $$r_{i}\left(\epsilon \right)=\frac{\left|M_{i}^{R}\left(\epsilon \right)\cap^{\text{​}}M_{i}\right|}{\left|M_{i}^{R}\left(\epsilon \right)\cup^{\text{​}}M_{i}\right|}$$

where $\cap^{\text{​}}$ and $\cup^{\text{​}}$ is the intersection and union operator, respectively, and |M| is the cardinality of the set M.

As shown in Equation (4), the higher r(ϵ) is, the better the recognition performance is. In this study, $r_{i}=\underset{\epsilon }{\max}r_{i}\left(\epsilon \right)$; was used as the recognition accuracy of the food image recognition method on the food category $C_{i}$. Table 3 represents the accuracy of the method with the various feature types. The accuracy was computed for each food category and for the overall food categories.

**Table 3:** Recognition accuracy of the food image recognition method with various feature types. SIFT, scale invariant feature transformation; LBP, local binary patterns.

	SIFT	LBP	Colour	SIFT + LBP + Colour
Beans	0.33	**0.53**	0.19	0.43
Carrots	0.37	0.44	0.46	**0.51**
Cheese	**0.16**	**0.16**	**0.16**	**0.16**
Custards	0.60	0.22	0.24	**0.62**
Milk	0.13	**0.78**	0.3	0.13
Muscle meat	0.40	0.32	0.5	**0.55**
Oranges	0.23	0.44	0.5	**0.57**
Peas	0.65	0.85	**1.0**	0.93
Tomato	**0.35**	0.29	0.29	0.33
Yoghurt	**0.54**	0.2	0.2	0.42
***Overall***	*0.37*	*0.42*	*0.38*	***0.47***
SIFT: scale invariant feature transformation, LBP: local binary patterns				

## 4. Discussion

A prototype of an automated dietary assessment on mobile devices has been described in this paper. Various challenges have been identified through this progressive step with future work continuing to address these challenges. Challenges faced were similar to existing studies [7] in this field with colour and multiple food items being of particular focus. As shown in Table 3, on average, the LBP outperformed both the SIFT and colour histogram and the SIFT gave the poorest performance overall. The combination of all features (SIFT, LBP and Colour) gave a better performance overall as anticipated due to the various advantages and disadvantages of each feature. One particular note was for the “Cheese” category, where the accuracy was low. This was likely due to the presence of other food types in the same image with cheese. The accuracy could be improved if the “Cheese” items could be captured at a closer distance, *i.e*., outliers and other food items other than cheese are not present in the image.

In the current implementation, linear SVMs were used. More sophisticated SVMs such as radial basis function (RBF) and polynomial kernel SVMs often gain better performance. Thus, those kernel types will be implemented, tested, and compared with the linear kernel in future studies. In addition, some advanced machine learning techniques, e.g., deep learning [22], extreme learning [23], will also be considered to improve the recognition accuracy. These techniques are recent developments in the area of pattern recognition.

Since multiple food types can co-occur on the same image, extracting individual food items would help to improve the recognition accuracy. Therefore, the aim is to simultaneously detect and recognise food as for future work. The accuracy of the food image recognition may also be improved if the classifiers were trained on large and diverse datasets including various illumination conditions, complex background, food images captured at various viewpoints*.* More challenging datasets with more food categories have been collected and will be released in our future work.

## 5. Conclusions

Applying image processing and pattern recognition techniques on a mobile device has allowed for the development of an automated food record via the use of a Smartphone. Continuing the prototype developed in this study to the subsequent stages of the food record, namely portion identification and translation to nutrient data, will complete the process and allow for a practical user-friendly approach to dietary data collection within the Australian context. It is vital that the mapping of food images to their corresponding food items within a food composition database is performed carefully to allow for the most accurate output data to be provided. Development of applications for the use in dietetic practice needs to encompass the inherent bias of the underpinning method of dietary assessment. They should also consider the advancements in technology to potentially reduce some of these biases. This will provide impetus for more robust dietary assessment processes that are streamlined in their methods but also less resource intensive in their nature. Considering these two concepts together will mean that nutrition researchers or clinicians in practice can spend additional time with the clients working on behaviour changes for better health rather than data entry and nutrient analysis as was previously the case. Embracing credible and suitably selected technologies to work within the existing nutrition care processes should be considered to the advantage of both the clients and clinicians. The work of this study is one of the first of this nature in the Australian context.
